# Pre-emptive Balloon Angioplasty for Asymptomatic Vasospasm to Facilitate Coil Embolization of a Ruptured Middle Cerebral Artery Aneurysm: A Case Report

**DOI:** 10.7759/cureus.88388

**Published:** 2025-07-20

**Authors:** Satoshi Horiguchi, Yoshinori Maki, Takeshi Kawauchi, Takeshi Satow, Taro Komuro

**Affiliations:** 1 Department of Neurosurgery, Nagahama City Hospital, Nagahama, JPN; 2 Department of Neurosurgery, Hikone Chuo Hospital, Hikone, JPN; 3 Department of Neurosurgery, Osaka Red Cross Hospital, Osaka, JPN

**Keywords:** aneurysmal subarachnoid hemorrhage, asymptomatic vasospasm, cerebral vasospasm, endovascular coil embolization, mca bifurcation aneurysm, percutaneous transluminal balloon angioplasty

## Abstract

Endovascular coil embolization of ruptured cerebral aneurysms during vasospasms presents technical and safety challenges. Although balloon angioplasty is typically reserved for symptomatic vasospasms, its use to facilitate endovascular procedures in asymptomatic cases has rarely been reported.

We present the case of a 45-year-old man with a four-day history of persistent mild headache. He had undergone coil embolization for left- and right-middle cerebral artery (MCA) aneurysms nine years earlier. On admission, non-contrast computed tomography confirmed a subarachnoid hemorrhage. Digital subtraction angiography revealed a newly ruptured aneurysm at the right MCA bifurcation, along with a segmental vasospasm extending from the right M1 to M2 segments. Although the patient exhibited no neurological deficits, balloon angioplasty was performed to dilate the narrowed artery, thereby facilitating safe microcatheter navigation and reducing the risk of thromboembolic complications during coil embolization. The aneurysm was successfully occluded, and the patient was discharged without any new neurological deficits.

This case suggests that, in select patients, balloon angioplasty may be considered to overcome anatomical challenges posed by asymptomatic vasospasm during endovascular treatment of ruptured cerebral aneurysms.

## Introduction

Cerebral vasospasm remains one of the most significant complications following aneurysmal subarachnoid hemorrhage (SAH), typically developing between days four and 14 after ictus [1, 2]. In cases where patients do not respond to medical therapy, intra-arterial vasodilators may be administered. Although effective for distal and diffuse vasospasms, their effects are often transient [3]. Balloon angioplasty is a well-established treatment for symptomatic vasospasm, demonstrating efficacy in restoring cerebral blood flow and preventing delayed cerebral ischemia [4-11]. However, the role of angioplasty in asymptomatic vasospasm remains controversial, as its prophylactic use lacks consensus and raises safety concerns [12, 13].

Endovascular coil embolization of ruptured aneurysms during vasospasms presents technical challenges [7]. Severe arterial narrowing not only complicates catheter navigation but also increases the risk of procedural complications such as arterial dissection or thromboembolism. In such cases, balloon angioplasty before coiling, despite the absence of clinical symptoms, may facilitate safe vascular access and reduce the risk of neurological deterioration.

Herein, we report a rare case in which balloon angioplasty was performed to treat an asymptomatic vasospasm, thereby enabling endovascular coil embolization of a newly ruptured middle cerebral artery (MCA) aneurysm in a patient with a history of SAH.

## Case presentation

A 45-year-old man presented with a four-day history of persistent, worsening headache. His medical history included a ruptured left MCA aneurysm. The initial attempt at surgical clipping via craniotomy failed because of severe brain swelling, which precluded access to the aneurysm. Endovascular coil embolization was subsequently performed, followed by embolization of the unruptured right MCA aneurysm one month later.

**Figure 1:**
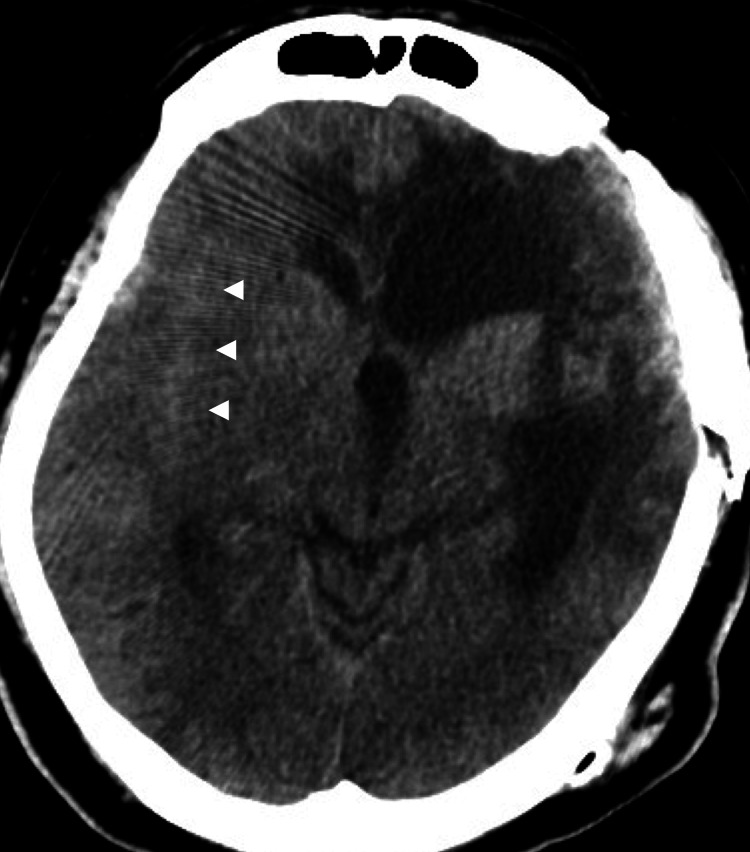
Non-contrast computed tomography (CT) on admission Axial non-contrast CT scan showing high-density areas within the right Sylvian fissure (arrowheads), consistent with a subarachnoid hemorrhage (Fisher grade 2).

Upon presentation, non-contrast head computed tomography (CT) revealed a subarachnoid hemorrhage classified as Fisher grade 2 (Figure 1).

**Figure 2:**
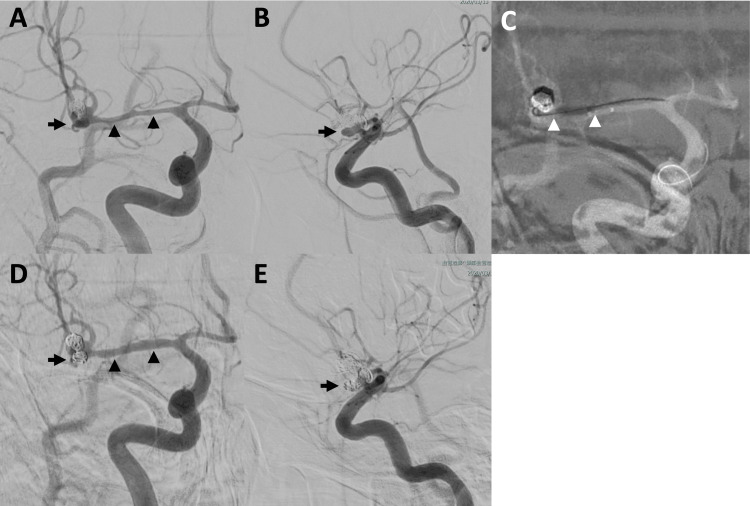
Intraprocedural digital subtraction angiography (DSA) images (A, B) Initial DSA in anteroposterior (A) and lateral (B) views reveal a newly ruptured aneurysm at the right-middle cerebral artery (MCA) bifurcation (arrows), along with segmental vasospasm from the M1 to M2 segments (A, arrowheads); (C) Balloon angioplasty was performed in the distal M1 segment. The proximal and distal ends of the balloon are indicated by arrowheads; (D, E) Post-procedural DSA in anteroposterior (D) and lateral (E) views demonstrates successful coil embolization of the aneurysm (arrows) and improved caliber of the right M1 segment (D, arrowheads) following balloon angioplasty.

The patient was alert and had no focal neurological deficits, corresponding to World Federation of Neurosurgical Societies grade 1 and Hunt and Hess grade 2. Digital subtraction angiography (DSA) revealed a newly developed saccular aneurysm at the right MCA bifurcation, measuring 4 mm in height and 3 × 3 mm in width (Figure 2A, 2B). Segmental vasospasm was observed in the right M1 to M2 segments of the right MCA. The M1 segment showed approximately 60% luminal narrowing.

Coil embolization was chosen because of the high surgical risk associated with the vasospasm period and anticipated adhesions resulting from the previous craniotomy. Although the patient exhibited no clinical signs of vasospasm, the arterial narrowing posed a potential obstacle to microcatheter navigation and increased the risk of thromboembolic complications during endovascular treatment. To mitigate these risks, balloon angioplasty was performed before embolization.

A compliant balloon catheter (SHOURYU 3-5 mm; KANEKA MEDIX, Osaka, Japan), originally prepared for possible neck remodeling and used as a hemostatic tool in case of re-rupture, was advanced to the distal M1 segment. Four sequential inflations were performed in a distal-to-proximal direction (Figure 2C). Post-angioplasty angiography confirmed an improved vessel caliber (Figure 2D).

Subsequently, coil embolization of the aneurysm was performed using an Excelsior SL-10 microcatheter (Stryker, Kalamazoo, MI, USA). Two detachable coils (Target360 Ultra 3 mm × 6 cm and 2.5 mm × 4 cm; Stryker) were successfully deployed without any complications (Figure 2D, 2E). The total procedure time was 60 min.

**Figure 3:**
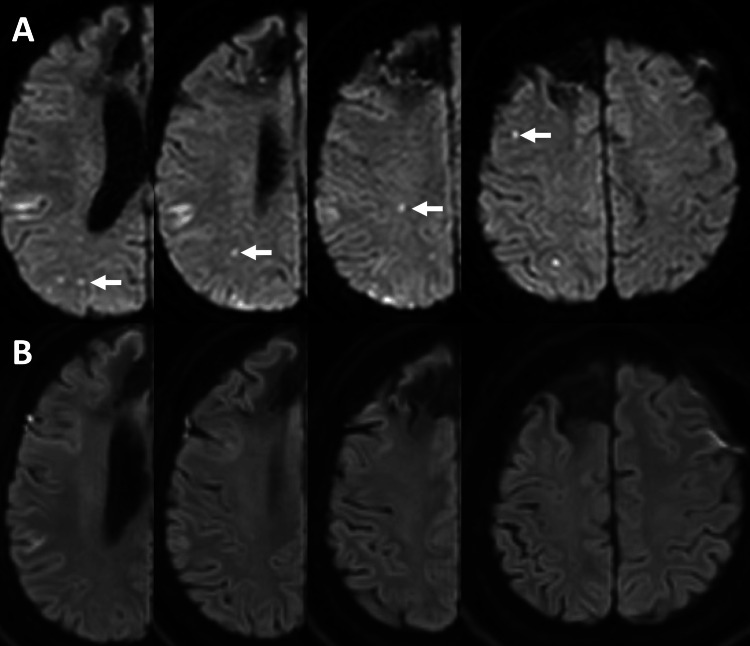
Postoperative diffusion-weighted magnetic resonance imaging (DWI) (A) DWI on postoperative day seven shows multiple punctate high-intensity lesions in the right cerebral hemisphere. Arrows indicate representative lesions; (B) Follow-up DWI on day 14 shows no new ischemic lesions

Postoperative magnetic resonance imaging (MRI) on day seven post-ictus revealed multiple punctate high-intensity lesions on diffusion-weighted imaging, each measuring up to 2 mm in diameter, and located in the right frontal, temporal, and parietal lobes (Figure 3A). These lesions may represent minor microembolic events or balloon-induced vessel irritation; however, they were asymptomatic. No new ischemic lesions were detected on follow-up MRI on day 14 (Figure 3B).

MR angiography revealed persistent visualization of the aneurysm neck with complete occlusion of the dome. The patient was discharged with a modified Rankin Scale (mRS) score of 2, consistent with his pre-hemorrhage baseline. The patient remained under outpatient follow-up for five years after embolization, with no evidence of aneurysm recurrence.

## Discussion

We report a case of SAH caused by rupture of a newly developed MCA aneurysm that was distinct from previously treated aneurysms. The patient presented with vasospasms, without any neurological symptoms. The ruptured aneurysm was successfully treated with coil embolization preceded by balloon angioplasty to safely dilate the narrowed right MCA M1 segment and facilitate endovascular access.

The treatment of ruptured aneurysms during the vasospasm period is associated with an elevated risk of complications. A sub-analysis of the International Subarachnoid Aneurysm Trial found that patients treated between days five and 10 after symptom onset had a 1.68-fold higher risk of delayed cerebral infarction than those treated within two days. Additionally, treatment beyond day 11 was associated with a 2.5-fold increased likelihood of an mRS score ≥3 at one year [14]. In patients who are clinically stable but at risk of rebleeding, safe treatment during the vasospasm window is essential.

Recent studies suggest that with appropriate patient selection and advancements in endovascular techniques, safe embolization during the vasospasm period is feasible. Koiso et al. reported successful coiling in patients presenting after day three, even in the presence of vasospasms along the access route [15]. Cho et al. described safe embolization facilitated by continuous intra-arterial nimodipine infusion to manage vasospastic segments [16]. However, balloon angioplasty was not performed in the previous cases. The present case is unique in this regard.

Balloon angioplasty is well documented as an effective treatment for symptomatic vasospasm [4-11]. However, its use in asymptomatic patients remains controversial. Eskridge et al. and Murayama et al. demonstrated its benefits in symptomatic cases but advised caution when applying it to vessels without clear clinical symptoms because of the risk of vessel injury [4, 6]. Recent evidence supports the potential role of balloon angioplasty and intra-arterial nimodipine infusion in severely narrowed vessels, regardless of the presence of clinical symptoms [11]. A recent review on the current management of SAH further recommends that balloon angioplasty should be considered early in all patients with severe angiographic vasospasm, even in the absence of neurological deterioration [17].

In this case, balloon angioplasty was deemed necessary to enable safe microcatheter navigation through the significantly narrowed M1 segment and to reduce the risk of thromboembolism or arterial dissection. This proactive strategy contributed to favorable procedural outcomes without complications.

This case underscores the importance of individualized treatment strategies, particularly in patients with complex medical histories and prior cerebrovascular intervention. Although routine prophylactic angioplasty is not advocated, carefully selected patients may benefit from this approach when the vascular anatomy poses a procedural risk.

## Conclusions

Balloon angioplasty for asymptomatic cerebral vasospasms may serve as a useful adjunct to ensure safe and effective endovascular treatment of ruptured aneurysms in select cases. This report adds to the limited literature supporting this strategy and suggests that when arterial narrowing threatens procedural safety, a proactive approach is warranted.
